# Leapfrogging medical infrastructure: a novel bilateral exchange model driven by digital medicine in Kazakhstan

**DOI:** 10.7189/jogh.16.03015

**Published:** 2026-07-03

**Authors:** Junichiro Takahashi, Kosuke Tanaka, Yosuke Sato

**Affiliations:** 1 Brain Function Analysis & Digital Medicine Research Institute, Showa Medical University, Tokyo, Japan; 2 Department of Neurosurgery, Showa Medical University Hospital, Tokyo, Japan

## Abstract

The geographic maldistribution of highly specialised neurosurgical care in Central Asia, particularly Kazakhstan, presents a critical challenge driven by medical education pipeline constraints and brain drain. Traditional assistance models relying on short-term missions or conventional hardware donations are insufficient to bridge this fundamental infrastructural gap. We present a novel ‘Bilateral Medical Exchange Model’ established between Showa Medical University in Japan and top Kazakh medical institutions. By utilising advanced digital medicine – specifically, high-performance computing-driven artificial intelligence and 3D extended reality visualisation – as the primary diplomatic catalyst, this framework successfully engaged both grassroots clinicians and top-tier government officials. We highlight how targeting these technologies toward localised capacity building and edge-computing infrastructure circumvents traditional barriers. While long-term clinical outcomes remain to be systematically evaluated, this technology-driven diplomatic approach provides a foundational, hypothesis-generating framework for bridging the digital divide, integrating digital innovation with decentralised education to ultimately improve equitable patient outcomes in transitioning economies.

Neurosurgical care requires highly specialised expertise, advanced diagnostic imaging, and robust infrastructure, making it one of the most resource-intensive medical fields. Globally, an estimated five million essential neurosurgical cases go unmet each year, disproportionately affecting lower-resource settings [1]. In Central Asia, particularly in Kazakhstan – an upper-middle-income country with the world’s ninth-largest land area – the geographic maldistribution of medical resources poses a critical challenge. The overconcentration of specialised neurosurgeons and high-performance diagnostic equipment in a few metropolitan centres leaves vast rural populations with limited access to equitable, high-tier neurological care [2].

The root causes of this access gap are deeply systemic, largely driven by constraints in the medical education pipeline and a continuous ‘brain drain’ of highly trained specialists from regional hospitals to urban centres. Traditional models of international medical assistance, which primarily rely on the physical dispatch of physicians, short-term surgical missions, or the donation of conventional hardware, are neither scalable nor sustainable for bridging this fundamental infrastructural gap [3]. While digital health innovations are frequently discussed as potential solutions [4], a significant gap remains in the literature regarding actionable frameworks that combine high-level diplomatic engagement with the immediate, localised educational deployment of these technologies.

Recent advancements in digital medicine – specifically, high-performance computing (HPC)-driven artificial intelligence (AI) and extended reality (XR) – have the potential to offer promising opportunities to bridge this divide. In concrete clinical workflows, AI can assist in rapid preoperative imaging interpretation, while XR can provide immersive 3D spatial navigation for surgical planning and remote anatomical training [5]. These technologies can help decentralise highly specialised surgical knowledge, allowing remote regions to access top-tier medical expertise instantaneously.

However, the true barrier to implementing such cutting-edge technologies in transitioning economies is rarely technological. It is deeply systemic, involving complex regulatory hurdles regarding medical data sovereignty, a lack of information technology infrastructure, and the absence of localised, tech-literate mentorship [6]. Therefore, technological innovation alone cannot achieve global implementation without a robust diplomatic and educational vehicle; it must be integrated with workforce investment and health system reform.

We present a hypothesis-generating ‘Bilateral Medical Exchange Model’ established between Japan (Showa Medical University) and Kazakhstan. Unlike conventional exchanges, this framework utilised advanced digital neurosurgical technologies as the primary catalyst. The core innovation of this model lies in utilising technology demonstrations not merely for clinical validation, but as a strategic tool to engage grassroots clinical practitioners and top-tier government officials simultaneously. We propose this framework not as a fully validated clinical intervention, but as a foundational step to explore how targeted digital health diplomacy might facilitate capacity building and initiate systemic healthcare modernisation in Central Asia.

## THE ‘SHOWA-KAZAKHSTAN’ EXCHANGE MODEL: TECHNOLOGY AS A CATALYST

The bilateral exchange between Showa Medical University and Kazakhstan’s top medical institutions evolved from foundational academic discussions into a strategic, policy-level partnership (**Table 1**). Initially catalysed by courtesy visits in 2023 with executives from the National Scientific Research Medical Center and the National Center for Neurosurgery, the collaboration reached a strategic engagement in late 2025.

**Table 1:** Typology and transferable components of the tech-driven Bilateral Medical Exchange Framework

Implementation phase and activity typology	Institutional and diplomatic prerequisites	Core governance mechanisms	Key objectives and technological focus
Phase 1: Foundation and protocol establishment	Shared awareness of regional healthcare disparities and mutual intent for bilateral collaboration	Direct engagement between institutional executives to establish foundational protocols	To identify broad clinical needs and define the scope of future digital health collaboration
Phase 2: Academic and clinical integration	Active engagement of frontline clinical leaders (*e.g.* Chief Professors of neurosurgery)	Sharing of advanced clinical insights and establishing frameworks for joint academic research	To align on specific sub-speciality challenges that require digital intervention
Phase 3: Targeted technology demonstration	Availability of scalable, proprietary digital tools addressing the identified clinical gaps	On-site demonstrations bridging grassroots practitioners, researchers, and students	To demonstrate the feasibility of specific tools (HPC-driven AI, 3D XR) for decentralised capacity building
Phase 4: Institutional formalisation	Validation of technological utility by partner institution leadership	Formal facility inspections and execution of a legally binding MOU	To secure a formal governance structure for collaborative research and localised technology testing
Phase 5: National-scale strategic planning	Escalation of the initiative from institutional to national policy levels	Strategic inspection of digital infrastructure by top-tier government and healthcare officials	To develop actionable roadmaps for national-level technology transfer and human resource development

AI – artificial intelligence, HPC – high-performance computing, MOU – Memorandum of Understanding, XR – extended reality

The breakthrough occurred during the International Conference of Neuroscience and Neurology in Astana (October 2025) and subsequent high-level meetings. Rather than focusing solely on traditional surgical techniques, our team demonstrated proprietary, cutting-edge digital medicine technologies developed at Showa Medical University. The demonstration centred on an AI-powered, real-time 3D visualisation system driven by HPC. This system converts 2D neuroimaging into interactive 3D spatial models, aiming to enhance spatial recognition for both pre-operative planning and intra-operative navigation.

The Kazakh medical leadership showed strong interest in the clinical potential of these tools. The delegation evaluated the technology not as an advanced surgical tool, but as a potential strategic approach to their geographical healthcare challenges. They recognised that the potential of this AI-driven 3D visualisation system to function as a remote, decentralised educational and diagnostic platform, standardising spatial anatomical training for regional surgeons while eliminating the need for relocation to the capital. The technological demonstration suggested the feasibility that high-level surgical expertise could be digitally packaged and distributed across their vast terrain.

This specific technological impact accelerated the diplomatic timeline. It directly led to the signing of a Memorandum of Understanding for joint research with the National Center for Neurosurgery. Furthermore, the resonance of this digital capability reached government officials. In December 2025, a delegation from the Medical Center of the President of the Republic of Kazakhstan – including the Deputy Director and the Head of Surgical Services – made an official visit to our institute in Tokyo. Their primary objective was the strategic inspection of our digital medicine infrastructure to discuss frameworks for national-level technology transfer and human resource development.

Thus, the proprietary digital technology served as a primary catalyst, transforming a conventional medical exchange into a dynamic model of digital health diplomacy. While these milestones represent significant progress in institutional collaboration, we acknowledge that the clinical and educational outcomes of this integration remain to be systematically evaluated.

## KEY BARRIERS AND NEEDS FOR IMPLEMENTATION

Through in-depth discussions with Kazakh healthcare executives, a clear consensus emerged: the primary driver for adopting advanced digital medicine is not merely academic interest, but a pressing clinical necessity. The Kazakh healthcare system faces a significant volume of neurosurgical patients, juxtaposed against a shortage of highly trained specialists and operational capacity. This imbalance highlights a significant workforce constraint, which serves as both a primary barrier to equitable care and a strong catalyst for digital transformation [7].

The delegation emphasised that their fundamental need is to utilise digital technology as a ‘force multiplier’ to bridge this human resource gap. However, transitioning these technologies from a Japanese research institute to the Kazakh clinical frontline involves overcoming several systemic barriers. To ensure actionable implementation, we have mapped these specific barriers to targeted technological mechanisms and prospective evaluation criteria:

1. The capacity and human resource bottleneck: The high volume of patients limits the time available for senior surgeons to mentor the next generation. Conventional surgical training is often too slow to meet the demographic demand [8]. To address this, our framework utilises 3D XR systems not merely as visualisation tools, but as decentralised training platforms. By engaging with immersive XR, junior surgeons can repeatedly simulate complex spatial anatomies in a risk-free environment, independent of senior surgeons' immediate availability. The measurable success criterion for this intervention will be tracking the time-to-competency in spatial anatomical recognition among trainees over a prospective 12–24-month evaluation period.
2. Infrastructural and edge-computing constraints: While the algorithms developed in Japan utilise HPC infrastructure, deploying these models in Central Asian regional hospitals presents hardware challenges. To handle the patient volume effectively, the required AI systems must transition from cloud-dependent models to edge-computing solutions. To operationalise this in practice, we propose deploying lightweight, optimised AI models directly onto localised edge-computing modules stationed at regional facilities. This allows for rapid, on-site image processing that bypasses the need for highly stable broadband cloud networks [9]. The expected outcome metric is a measurable reduction in diagnostic turnaround times for complex neuroimaging in participating rural hospitals.
3. Regulatory and data governance hurdles: To continuously optimise the AI models for the local population, the cross-border sharing of clinical data and the establishment of unified data governance standards are necessary. Navigating the regulatory frameworks regarding medical data sovereignty in emerging economies remains a complex hurdle. To overcome this, our model leverages the formal Memorandum of Understanding to establish secure, anonymised data corridors compliant with both nations' legal frameworks [10]. Success will be indicated by the authorised execution of initial cross-border pilot datasets for model fine-tuning within the next two years.

Addressing these barriers requires more than commercial technology transfer; it necessitates a comprehensive policy approach where digital tools are integrated directly into national medical education and infrastructure planning.

## POLICY RECOMMENDATIONS AND FUTURE DIRECTIONS

To successfully scale the Japan-Kazakhstan model across Central Asia and other emerging economies, a paradigm shift in international medical cooperation is required. We propose the following policy directions:

First, bilateral medical exchange should transition from the fragmented export of commercial hardware to the deployment of integrated ‘technology-and-education’ ecosystems. Digital health diplomacy should utilise academic institutions and cultural hubs – such as cross-border medical associations – to facilitate not only the transfer of algorithms but the continuous, localised training of personnel. To implement these shifts across diverse healthcare systems, policymakers must conduct localised needs assessments to ensure digital tools are directly integrated into existing national medical curricula.

More fundamentally, global health policies should realign the objective of digital medical development. Currently, the narrative surrounding medical AI is frequently dominated by commercial profitability and administrative workflow efficiency. However, the core purpose of digital innovation in healthcare should remain firmly oriented toward improving patient outcomes. Advanced technologies like HPC-driven AI and XR are clinical tools designed to mitigate geographic and systemic disparities.

When digital tools are deployed with this patient-centred focus, they can become the foundation upon which healthcare professionals refine their expertise, ultimately fostering the evolution of a more equitable, cross-border medical culture. Policies should be drafted to ensure that digital health funding and cross-border data governance frameworks are aligned with this philosophy.

### Limitations

With this viewpoint, we present a preliminary, hypothesis-generating framework, and several limitations must be acknowledged. First, there is currently an absence of long-term, patient-level outcome data and quantitative training metrics to validate the clinical efficacy of this model. The milestones achieved thus far represent early-stage institutional engagement rather than demonstrated clinical improvements. Second, the replicability of this model in other lower-resource settings may be challenged by barriers related to diplomatic reach and institutional resources. Kazakhstan, as an upper-middle-income country, possesses a baseline academic and technological infrastructure that may not exist in lower-income nations. Implementing this framework requires substantial initial investments in HPC, localised edge AI infrastructure, and the establishment of formal bilateral diplomatic relationships. Future studies must prospectively evaluate the cost-effectiveness, educational outcomes, and direct impact on patient care to empirically validate this tech-driven diplomatic model.

## CONCLUSIONS

The bilateral exchange between Showa Medical University and Kazakhstan demonstrates that advanced digital medicine can serve as a valuable diplomatic catalyst. High-performance AI and 3D XR technologies have the potential to help bypass traditional infrastructural barriers, delivering top-tier neurosurgical expertise to regions suffering from extreme geographic and human resource maldistribution.

By utilising technology as a catalyst, this model engaged top-level government officials and frontline surgeons alike, establishing a foundational framework for capacity building. Ultimately, while the long-term clinical impacts remain to be systematically evaluated, bridging the digital divide in neurosurgery requires a unified commitment to integrating technological advancement with localised education, aiming to improve patient outcomes and elevate the global culture of medicine.
